# State of Lunacy in Ireland

**Published:** 1857-10-01

**Authors:** 


### Art. VII.—STATE OF LUNACY IN IRELAND.

The Eighth Report of the "District, Criminal, and Private Lunatic Asylums in Ireland" for 1857, presented by the Commissioners to the Houses of Parliament, has been forwarded to us through the politeness of Dr. Nugent, who, owing to the illness of his colleague, Dr. White, has almost the sole responsibility of this Report. It is most ably and comprehensively constructed, and contains matter of the deepest interest, not only in relation to the state of lunacy and lunatics in Ireland; but as suggestive of many valuable considerations in reference to the relations which unsoundness of mind bears to the administration of justice in general; as also many other topics bearing directly upon the great social questions which are now attracting much attention, chiefly as to criminal responsibility—education, secular and religious—domestic life, &c. A more favourable contrast can scarcely be imagined, than is presented between the aspect of lunacy in general, as treated of in this Report, and that which has recently excited so much and so indignant comment as existing in a sister country. We purpose to condense the substance of this document, so as to give a full view of the important matters treated upon.

In March, 1855, there were in Ireland of lunatics, idiots, and epileptics, 13,493, of whom 6263 were under official supervision in asylums, public or private, gaols and poor-houses. In March, 1857, the number was 14,141 altogether; of whom 6520 only were located in public or private institutions. In both cases, above half the entire number were at large, and are described by Dr. Nugent as "being possessed of means of their own, supported by their friends, or wandering from place to place, depending for a precarious subsistence on the charity of individuals."

This seems to be a considerable proportion of the insane to be at large. In examining into the causes, it would appear that notwithstanding the very large outlay which has been expended in the erection of district asylums in the various counties, the accommodation is still very far below the requirements; and as will shortly appear, the number confined in private establishments bears an extremely small proportion to the total. Altogether, upwards of 600,000*l.* has been expended for these purposes, "including land, buildings, furniture, and fittings;" yet there is accommodation in these buildings for only 4337 persons. In March, 1857, there were 3856 patients in these establishments, leaving only 481 beds unoccupied throughout the country; not a large margin for contingencies, it must be acknowledged. Some of the largest houses, as that at Richmond, had only three or four vacant; by no means sufficient for the constantly expected additions, and the uncertain discharges.

Looking at the entire numbers of lunatics, &c., quoted by Dr. Nugent, as at present existing in Ireland, we are somewhat surprised at the low amount. In England and France the average number of persons of unsound mind, including epileptics, &c., is about 2 in every 1000.* If we calculate upon the same proportion for Ireland, the number of lunatics in 1855 would only account for a population of 6,746,500—certainly below the real number. Whether lunacy be actually less in Ireland than in our own country, or whether there may not be some unexplained deficiency in the returns, we are not prepared to say. As to the accuracy of the information, Dr. Nugent observes, speaking of the lunatics that are not under official surveillance:—

"Of these latter we have again obtained very valuable returns through the constabulary, and from the extensive distribution of that efficient force throughout the country, combined with the careful manner in which the returns have been prepared, we think the information may be relied upon as accurate, the more so as we improved on the forms previously used, having now got the name, age, address, and religion, of every individual in Ireland, whether lunatic, idiot, or simply epileptic."

The number of epileptics is 2171, concerning whom the Report observes—"We do not mean to say that supervision is generally required, for save during the temporary attacks of a paroxysm, they are for the most part perfectly competent to take care of themselves."

With regard to those under treatment during the two years embraced in this Report, the recoveries were 17 per cent., and the *relieved* 11½ per cent.

"The mortality, 9 per cent., is just one below that of the preceding biennial period; and when the proportion of unpromising cases brought

* On the authority of MM. Lélut and Tardieu.

from prisons is taken into account, these facts speak most favourably of the successful issue of Irish asylums. No epidemic of any kind has visited them since June, 1853, when we had occasion to refer to an increased number of deaths at Belfast and elsewhere from cholera. The mortality is now, for the most part, referable to affections of the brain and nervous system, or to diseases associated with organic debility, but particularly of the lungs, and ending in consumption. Two cases were suicidal; the first occurred at Belfast, in 1856, a male patient accomplishing his object by suspending himself from the ventilator in one of the single sleeping rooms. The second was at the Richmond, where a patient strangled himself at night with a sheet taken from his bed, which he tore up for the purpose. The coroner was called in on both cases, and it appeared on inquiry that neither of the lunatics had ever evinced dangerous tendencies, nor had the physicians or immediate attendants any suspicion that they meditated self-destruction. These, with another instance in which an old man was suffocated whilst eating, were the only casualties of a fatal nature which occurred during the period embraced in this Report."

As to the treatment generally adopted in the district asylums, it does not appear that there is any special course adopted, except in recent cases, or when marked symptoms require it in those of older standing. "Air, regimen, exercise, with the removal of causes leading to excitement, being more favourably regarded as tending to a beneficial result, in the alleviation of a malady, as yet but imperfectly understood."

On the subject of mechanical restraint, queries were addressed to the managers of the district asylums, and the result is as follows:—

"With reference to physical coercion, or mechanical restraint, in the majority of asylums it is employed in a mitigated form, in the others it is seldom or never had recourse to. On this mooted subject we do not interfere, unless, as has occasionally happened on inspection, we considered that the appliances might be partially if not altogether discontinued. The question is one more properly for the judgment of the local medical superintendents, and as we believe they are alike influenced by the most humane motives and a desire to do what is best for the safety of their patients, we would deem it unadvisable on our part to lay down any fixed rule on a system which is at issue between enlightened practitioners, and which, further, there is no authority to enforce. In our opinion, however, one most urgent and almost insurmountable objection exists to mechanical restraint, and which arises from the contingency of its being surreptitiously employed by attendants, to avoid trouble, unless due precautions are taken by the resident physician or manager."

The remarks upon religious ministrations and education appear to us of sufficient importance to quote at length:—

"With reference to religious ministrations—a subject which, unfortunately, has given rise, within the last three or four years, to a marked difference of opinion between the executive and the governors of a northern asylum, the majority of whom, actuated, no doubt, by the sincerest motives, successfully opposed the admission of officially appointed chaplains—our sentiments, far from undergoing any change, have been strengthened by daily experience. We have specially directed ourselves to the points at issue, personally attending in asylums, at the respective hours of public worship, questioning patients themselves, inquiring both of officers and attendants, and noting the results at the moment; and we cannot arrive at any other conclusion, than that the regular visitations of chaplains, and the due performance of divine worship, should not be denied to the inmates of public institutions for the insane; for apart from other and higher considerations, the soothing influence of religion, as tending to the establishment of a self-control, however temporary in its nature, cannot but be valuable in a curative point of view; and it should not be forgotten that, though in one individual the reasoning powers are normally affected, the sentiments may remain unchanged, whilst in another, the moral feelings may be deranged, at the same time that the intellectual faculties are comparatively unimpaired—both cases being alike susceptible of the benefits of religion.

"We have made an analysis of the state of education of the insane in poorhouses and asylums. In the former, it appears there are 323 more or less educated, and 1476 illiterate; in the latter, the numbers are 2353 with some degree of education, against 1505 totally ignorant. The proportion of literate to illiterate in the general population of this country is fifty-three per cent. If we restrict the comparison to those in asylums, omitting the inmates in poorhouses, of whom a large majority are idiots, it would appear that education is in a much higher ratio among lunatics than in the community at large—a circumstance indicative of the fact that insanity, even among the humbler classes, is connected with intellectual development. At the Richmond, a school was established about six years ago, principally for females of weak mind, and though not likely to be followed by any permanent result, has at least the merit of a benevolent intention, and tends to vary the daily occupations. We would therefore wish to see the example of the Richmond governors adopted in other establishments, and also a more liberal supply of some cheap periodicals afforded."

With reference to the opinion here expressed, that insanity is connected with intellectual development, it may be well to examine with some care into the statistics, though this is not the place to enter into the question fully. Out of 3856 patients under treatment in the district asylums in March, 1857, 236 are reported as "well educated;" 516 can read and write "well;" 821 can read and write "indifferently;" 778 can read only; 1505 cannot read. It may be urged and granted that intellectual development is not always in strict proportion with education; but if we judge from these figures alone, they would scarcely appear to support very strongly the view here taken.

On the causes of insanity, Dr. Nugent thus writes:—

"Hereditary predisposition and intemperance would seem to be the two great feeders, if the term may be used, to lunatic asylums. In an aggregate of 3856 individuals, on the 31st March, we find of the 2146, where causes are assigned, no less than 997 under these denominations, 506 of the former, 491 of the latter, or forty-six per cent. As regards the cases where we had no definite information—and these are constituted, for the most part, of transferences from gaols—it is legitimate to conclude that the same proportion as in the assignable exists. Hence of the whole population in asylums, 1790 come within the two categories. This fact alone, pregnant of serious considerations, speaks for itself, and needs no comment on our part.

"Under the head of exciting causes to insanity, religion is enumerated; but considering the great influence which it exercises over the conduct of mankind, not alone for good, but unfortunately too, from a misconception of its true spirit, imbuing whole communities occasionally with a disposition to commit the wildest acts from the most unreasonable motives, it does not seem to be so powerful an agent in producing individual mental derangement as might be at first supposed. Lunatics will, no doubt, readily adopt, and as quickly abandon, extravagant ideas on religious as on other subjects, whilst the really exciting causes will be found totally unconnected with them. We find among clergymen and the members of pious associations more perhaps than an average per centage of lunacy; but their delusions rarely refer to their previous avocations, an observation alike pertinent to the insane members of other professions. Love, from misplaced affections and disappointed hopes, is a much more fertile source of the disease, particularly among the female sex, who, from their habits and sensibilities, are more susceptible than men of those influences recognised under the designation of moral."

On the increase of insanity, Dr. Nugent makes allowance for the increased facilities for detection and enumeration as augmenting the annual returns; but at the same time—

"The important truth must not be overlooked, that from each individual case of lunacy, germs of disease, to be developed at a future period, possibly in a third or fourth generation, may be produced; for such is our organization, that the mind no less than the body partakes of inherent and hereditary peculiarities, which, as your Excellency is aware, rendering nations at large distinguishable by corporeal prowess, valour, progression in the arts, &c., or the reverse, first find their way into the smaller circles of which those nations are composed.

"'Quia multa modis primordia multis
Mista suo celant in corpore sæpe parentes
Quæ patribus patres tradunt a stirpe profecta.'"

The Commissioners consider that on the whole the district asylums of Ireland have been successful, both in a curative and protective point of view; that they are well managed, and that the domestic arrangements are generally good, though perhaps inferior to many of the English, still more to *some* of the French asylums.

"Generally speaking, a deficiency of furniture, and with it a certain air of discomfort, is noticeable in Irish institutions for the insane, a want which we trust, with the advancing prosperity of the country, will be gradually obviated; yet, when your Excellency, so long and thoroughly cognizant of the social condition of the population, recals to mind what on your frequent visits to District Asylums you could not fail to remark, and reflects that a large proportion of their inmates whilst possessed of reason, had been strangers to the personal comforts of life, and, we regret to add, in many instances, from their abject state of destitution, to the decencies of civilization, but still protected by an innate sense of virtue and decorum—huddled together in those miserable abodes which present themselves in quick succession along our public thoroughfares, on the edge of bogs and sides of mountains—without adequate food or raiment—whole families frequently occupants of a single apartment, perhaps of a common bed—that the same individuals placed in asylums, labouring under madness in all its varied forms, are educated for the first time to habits of order and cleanliness, have servants at all hours to minister to their personal wants—their dress and bedding duly attended to, meals served regularly with a liberal allowance of animal food, a luxury before almost untasted by them—we may, as tending to social advancement no less than for curative objects, so far regard our public establishments for the insane with unmixed satisfaction."

The Commissioners find much fault with the system of committing "dangerous lunatics" to gaols; the law in Ireland being essentially different from that in our own country. The magistrates are empowered to imprison persons "discovered under circumstances denoting a derangement of mind, and a purpose of committing an indictable offence;" and from prison they may be transferred to the district lunatic asylum by a warrant of the Lord Lieutenant. The number so committed during the last two years amounted to 1296.

The objections are, that gaols become thereby only "so many channels of transmission to lunatic asylums, whereby a serious derangement "of prison discipline and considerable additional expense to the public are produced." The Commissioners have before commented upon this; but the abuse seems to have increased. It operates unfavourably in another respect, that in this manner families get rid of their troublesome insane members in a summary manner. They scarcely think it worth while to apply for an admission to the asylum in the ordinary manner, but they "depose to the existence of violent tendencies, the *result too often* of premeditated irritation." Under the guard

* 1 Vict. cap. 27.

of the police, the unfortunate individual is conveyed away to the county prison, and "with this act the curtain drops between the parties."

"The usual form of admission into a District Asylum requires an attestation as to the residence, birth-place, and social state of the person desired to be admitted; also an engagement on the part of some responsible party to take back the patient when called upon to do so. A committal obviates all this responsibility, and we have frequently known of applicants who were refused admission by the local board on sufficient grounds—such as not being natives of, or in any way connected with the district—having been sent to gaol as dangerously insane, and in this manner ultimately forced on the institution. Thus, while admitting that the Act was in some measure necessary, we cannot shut our eyes to the many and glaring abuses which have arisen from it.

"Of the conduct of the class in question, we find that the great majority, far from exhibiting any of the dangerous tendencies attributed to them, are tranquil and amenable from the very moment they are placed in confinement; and, generally speaking, this character attaches to them afterwards when transferred to asylums—the so-called 'dangerous,' however troublesome, evincing as little propensity to violence as ordinary lunatics."

Very few instances appear to have occurred of anything approaching to cruelty in the treatment of those thus committed; and Dr. Nugent even considers that as to recovery, there are elements of success met with in gaols, which are not found to the same extent in the asylums; such as more frequent association with the sane, and the fact that if thus committed, it is generally in the earlier stages, and therefore the affection is more amenable to treatment.

The notice of the Central Criminal Asylum at Dundrum is especially interesting from the nature of its cases, and from the reflections on the subject of criminal responsibility which it suggests. The number of criminal lunatics remain exactly the same as at the last report—82 males and 44 females. In the two years 26 have been discharged or died. Of these—

14 cured—7 liberated by order of the Lord Lieutenant.
3 sent back for trial.
3 sent back to gaol.
1 sent to a county gaol.
1 removed to a private asylum.
11 died.

With reference to insane convicts, the Report speaks as follows:—

"The greatest, indeed the sole, difficulty to be dealt with at the Central Asylum arises from the occasional residence in it of culprits who either feign insanity or whose claim to the designation of lunatic is at best but doubtful. There are persons transferred from gaols subsequent to conviction, and at whose trial the question of insanity was never entertained. Some of these may be faithfully described as having set discipline at defiance. Indifferent alike to remonstrance and to punishment—influenced, as it were, by a determination not to yield till their object was attained—intractable in prison—their conduct uncertain and unaccountable—the authorities there report to the Executive that they have no appliances for their treatment; that the establishment is kept in turmoil and confusion by such characters; and submitting their removal to the Criminal Asylum, to which they are accordingly drafted as vacancies occur.

"The air of Dundrum, the situation, remarkable for its cheerfulness and salubrity—transition from close confinement, and the restraint and regimen of a prison, to comparative freedom and a better diet, produce in a short time a change in their demeanour; they still, however, consider themselves entitled to be supported by Government; and that being recognised as lunatics, they should not be required to work."

A considerable number of the committals to Dundrum appear to the Commissioners to be of questionable propriety, as may be observed in the following passages:—

"The question hence arises, are persons to be recognised as lunatics by law, who, thoroughly cognizant of right and wrong, having committed offences against public order, and whilst undergoing the punishment consequent thereon, set authority and discipline at nought by their insubordination and perversity of temper? For if, after conviction, these traits of character protect from the penalties attached to crime, they should, *a fortiori*, at trial, when duly established, procure acquittal on the plea of mental incapacity, although the parties may be competent to distinguish good from evil. The solution of this point is a matter of great importance, not alone to the criminal code of this country, but likewise to that of the sister kingdom. . . . .

"During the last ten years we have had a tolerably large experience in lunacy, feigned as well as real, and in aiding the prosecution of certain capital cases to conviction, have, with local physicians, been instrumental in checking it as a plea; to those cases we have cursorily referred in previous Reports. The benevolence certainly—possibly, it may be, the superior knowledge—of some would associate crime with insanity; we do not, however coincide in the view, that a disregard of moral perceptions can qualify deeds, the results as well as the responsibilities of which are perfectly well understood beforehand by the perpetrators of them; at the same time we cannot but consider it a misfortune to the insane to be acquitted on the plea of lunacy, without that special statement of every circumstance which might tend to establish their irresponsibility, or mitigate the character of their offence; in either case, acquittal often tells against the parties themselves, changing a definite to an indefinite period of confinement, a fact most justly suggested as a precaution to counsel, we believe, by the late Baron Alderson; but it is not with the legal so much as the social point we have to deal; for in the asylum at present there are two or three inmates confined *as lunatics*, who never evinced a symptom of insanity to our knowledge; one of whom particularly inveighs against having been transformed into a lunatic in the dock by counsel, to his great dismay and surprise at the moment, and to his continued discontent for a period of over eight years, during which he has been in confinement."

The Commissioners speak in very high terms of the judicious and benevolent management of Dr. Corbet, the resident physician, under whose government, without mechanical restraint, and without punishment of any kind, violence is almost unknown; and the transgressions are rarely more serious than the breaking of a pane of glass. The patients are generally occupied, either in their previous trades, in the farm, or in some other appropriate employment.

In the private asylums the number of patients is 462—252 males and 210 females: 91 have been discharged, 47 cured, and 44 "very much improved."

The sanitary condition of these private establishments is said to be excellent, the mortality being only about 4 per cent. on the entire number. This compared with the estimate given by the authorities on the statistics of insanity would appear to be remarkably low. Dr. Thurnam says that whilst a mortality of more than 9 or 10 per cent. must be considered high, one less than 7 per cent. is highly favourable.

Concerning mechanical restraint there is the same variation as in the public institutions—in all cases, however, it is very slight, in some it is entirely relinquished under all circumstances. The Commissioners, after relating one or two cases illustrative of the various views and requirements, add:—

"On this subject we may observe, that the total abolition of mechanical restraint has been strongly advocated by some of the most eminent physicians both in England and in this country; while, as we have already stated, others of great practical experience, contend that there are cases, such as the above, in which it cannot be wholly dispensed with, without imminent risk to the patient, and possible injury to the attendants. We cannot but think that, at least, the discussion of the question, and the support given to the non-restraint system, have been of incalculable advantage, inasmuch as it is now acknowledged, that on most occasions where recourse would heretofore have been had to mechanical appliances—perhaps in a severe form—they can now, with safety, be entirely dispensed with; and that only in extreme cases, where the physician must be left to the free exercise of his own discretion, should restraint of any kind be resorted to."

The following remarks on Moral Insanity and on Hereditary Transmission are interesting and instructive:—

"As formerly, so within the past year, cases of moral madness, originating in drink and dissipation, have been frequently admitted into private licensed houses. Some of them, discharged after a few months' confinement, were not since re-admitted, whilst others have been brought back. These latter cases are most perplexing: even after the lapse of a few days the salutary effects of control are visible in their regard; once free, however, they become the mere children of impulse, reckless of personal respect, regardless of the value of money, and scorning even decency itself. Rational in conversation, and most plausible in manner, within the asylum, their conduct is displayed out of doors in a series of the most irrational actions. It is painful to keep such parties confined, but still more so to let them run at large to certain destruction. As an illustration, we may adduce the case of a lady, at present in confinement in a private licensed house, who has been admitted and discharged four or five times within our knowledge, and who, when at liberty, and mistress of her allowance, spends it in one continued orgie of drink and dissipation. We may here observe, that a characteristic of this class is, at all times, an utter disregard of truth, together with an unceasing desire to impose upon the credulity of their hearers by the most specious pretensions to sense and wisdom, and the most solemn promises that a future morality would efface the errors of their past life.

"In the course of the year just elapsed, we have observed some instances strongly illustrative of the hereditary transmission of lunacy, and the extent to which it runs in families, so many as four relatives, in the degree of parent and child, having been confined in an asylum together—a fact fraught with serious consideration, and involving even the prospective position of the unborn themselves. Two, and even three members of the same immediate family labouring under symptoms of insanity is, to our knowledge, a matter of common occurrence. At Swift's Hospital, for example, now in existence for more than a century, we find, since its foundation, the same stock to have been continuously represented by its insane members."

We give the general summary of the Commissioners, with their recommendations, entire :—

"Summing up the results of our progressive experience from year to year, on the state and bearing of lunacy in this country, and on the organization of establishments generally for the insane, we would recommend that the existing asylums, repaired fully where repairs are needed, and supplied with the appurtenances of judiciously constructed establishments, such as lavatories, baths, workshops, water-closets, &c. &c., should be constituted hospitals for the treatment of mental disease in its early and more curable stages, as well as receptacles for lunatics, no matter how hopeless their recovery, but who, for their own or public safety, require a constant and careful supervision. For these two legitimate objects, we think, with similar establishments in Donegal and Wexford, and certain enlargements at Clonmel and Armagh, the present asylums could be rendered amply sufficient for the exigencies of the country. Most of the original institutions require to be partially remodelled, to secure a better classification of patients; and as they are, with one or two exceptions, deficient in infirmaries, chapels, laundry accommodation, and in large halls or dining-rooms, suitable provision under these heads should not be overlooked. We would disembarrass poorhouses of all lunatic inmates, and existing asylums of the idiotic and epileptic classes, who are at present supported therein at a large expense and without any commensurate hope of benefit, by allocating them in plain structures, but still well devised for the object, where quiet and chronic patients might find a refuge, placed under the same central control as the acute and dangerous just referred to, with a scale of dietary and social comforts beyond what are conceded to the ordinary paupers of a union, having ground for exercise and employment, and their support provided for as in the primitive houses by quarterly advances from the Treasury. For this object we do not think that provincial depots would be successful: the number of counties attached to each would cause much embarrassment in their working—and the unavoidable expense and inconvenience consequent upon the conveyance of patients from remote localities constitute serious objections. It appears to us more feasible, that each district requiring one should have a chronic hospital for itself; and if the area was extensive, such as that connected with the Ballinasloe Asylum, it might be matter for consideration whether it would be more judicious and beneficial to the counties to have the buildings in question at a distance from, or adjoining the parent establishment.

"Of the present system of management through Governors, subject to certain modifications, on the whole, we cordially approve; believing that gentlemen of education, rank, and position, whose acquaintance with fiscal affairs, and personal interest in the well-being of the country, afford the best guarantee for the due and economical expenditure of its rates, are the most suitable persons to appreciate the peculiar claims of the insane on the benevolence and liberality of the public; as well as from the fact, to which we can personally bear the most gratifying evidence, that a question of party or religion was never mooted within the walls of an Irish Asylum."

In the copious Appendices to this Report there is a vast mass of statistics, from which we extract a few details.

The total number of patients that had been treated in the district asylums during the year 1856-7, from March to March, was 4888. Out of this number 542 had been cured, 149 "improved," 45 discharged "not cured," 1 "incurable," 4 had escaped—whilst 3856 remained under treatment. The deaths amounted to 291, of which 290 were from "natural causes," one only accidental (suicidal).

The causes of death are worthy of note, consumption being by far the most frequent. For the two years ending March, 1856 and 1857, there were 559 deaths, the chief cause as follows:—

| | M. | | F. | | Total. |
|---|---|---|---|---|---|
| Consumption | 48 | ... | 69 | .. | 117 |
| General debility and old age | 41 | ... | 49 | ... | 90 |
| Paralysis | 40 | ... | 13 | ... | 53 |
| Epilepsy | 34 | ... | 10 | ... | 44 |
| Dysentery | 21 | ... | 14 | ... | 35 |
| Marasmus | 16 | ... | 15 | ... | 31 |
| Apoplexy | 20 | ... | 10 | ... | 30 |
| Pulmonary and bronchial affections | 12 | ... | 19 | ... | 31 |
| Dropsy | 1 | ... | 9 | ... | 10 |

It will be remarked how very disproportionate are the male and female deaths from apoplexy, epilepsy, and paralysis; the balance being kept up by the greater number of deaths from consumption and all other pulmonary affections; also in dropsy The other causes of death are comparatively unimportant.

An investigation of the trades, &c., of the inhabitants of the public asylums gives curious results. Out of the 3856 under care in March, 1857, no less than 1465 were connected with agriculture; 367 were domestic servants, only 42 of them males; 88 were dressmakers; 58 shoemakers; the other handicrafts about 40 each.

Under the head of "miscellaneous occupation" are found 239, only 21 being male;—"unknown" are 273 male and 638 female.

The forms of disease are thus given:—

| | |
|---|---|
| Mania | 2102 |
| Monomania | 208 |
| Dementia | 574 |
| Melancholia | 580 |
| Imbecility and epilepsy | 216 |
| Idiocy | 176 |
| Total | 3856 |

The *causes* are classed as—

| | | | M. | F. |
|---|---|---|---|---|
| Moral | 692 | ... | 245 | 447 |
| Physical | 948 | ... | 614 | 334 |
| Hereditary | 506 | ... | 231 | 275 |
| Not known | 1710 | ... | 879 | 831 |

Amongst the moral or mental causes, grief (210) and reverse of fortune (184) are much the most frequent. Love and jealousy furnish 97 cases, of which 70 are females; terror 79, of which 53 are females; religious excitement 70, 48 being females; domestic quarrels 15, 12 being females.

Of the physical causes, intemperance (491) and epilepsy (150) represent above two-thirds of the cases; after these, fever and bodily injury are the most frequent. Injury of the head is only 36 times the cause.

Dr. Nugent gives a table indicating the probability of cure as applied to these 3856. He supposes 1187 to be curable, 2222 to be probably incurable; 194 are idiots, and 253 are lunatic epileptics.

There is something instructive in examining the account of the previous occupations of the upper classes of the insane, those, viz., who are confined in private establishments. The entire number amounts to 462, out of which 253 had *no occupation.* The remainder were thus divided: army 23, navy 5, church 24, law 20, medicine 7, students 14, trade 37, farmers 29, other occupations 50.

With the exception of monetary matters, we have now passed in review all the most important subjects treated upon in this very valuable Report, which, however, will well repay a detailed perusal.

---

